# Strategies to address COVID-19 vaccine hesitancy in First Nations peoples: a systematic review

**DOI:** 10.1080/16549716.2024.2384497

**Published:** 2024-09-04

**Authors:** Adeline Tinessia, Katrina Clark, Madeleine Randell, Julie Leask, Catherine King

**Affiliations:** a School of Public Health, Faculty of Medicine and Health, University of Sydney, Sydney, Australia; b National Centre for Immunisation Research and Surveillance (NCIRS), Kids Research, Sydney Children’s Hospital Network, Sydney, Australia; c Population Health - Hunter New England Health, Wallsend, Australia

**Keywords:** Indigenous populations, vaccine interventions, vaccine equity, mistrust, vaccine hesitancy

## Abstract

**Background:**

First Nations peoples face disproportionate vaccine-preventable risks due to social, economic, and healthcare disparities. Additionally, during the COVID-19 pandemic, there was also mistrust and hesitancy about the COVID-19 vaccines among First Nations peoples. These are rooted in factors such as colonial histories, discriminatory medical practices, and unreliable information.

**Objective:**

To examine strategies to address COVID-19 vaccine hesitancy among First Nations peoples globally.

**Methods:**

A systematic review was conducted. Searches were undertaken in OVID MEDLINE, OVID EMBASE, OVID PsycINFO, CINAHL, and Informit. Searches were date limited from 2020. Items included in this review provided primary data that discussed strategies used to address COVID-19 vaccine hesitancy in First Nations peoples.

**Results:**

We identified several key strategies across four countries – Australia, the USA, Canada, and Guatemala in seventeen papers. These included understanding communities’ needs, collaborating with communities, tailored messaging, addressing underlying systemic traumas and social health gaps, and early logistics planning.

**Conclusion:**

The inclusion of First Nations-centred strategies to reduce COVID-19 vaccine hesitancy is essential to delivering an equitable pandemic response. Implementation of these strategies in the continued effort to vaccinate against COVID-19 and in future pandemics is integral to ensure that First Nations peoples are not disproportionately affected by disease.

## Paper context

**Main findings:**

In this review, we identified seventeen studies detailing five key strategies to reduce COVID-19 vaccine hesitancy among First Nations Peoples.

**Added knowledge:**

First Nations-centred strategies to reduce COVID-19 vaccine hesitancy were: understanding communities’ needs, collaboration with communities, tailored messaging, logistics planning and addressing the underlying systemic trauma experienced by First Nations peoples when accessing healthcare.

**Global health impact for policy and action:**

First Nations-centred strategies must be included in the continued effort to vaccinate against COVID-19 and other future outbreaks to ensure that First Nations peoples are not disproportionately affected.

## Background

Management of COVID-19 was contingent upon ensuring high vaccine uptake to minimise serious illness, hospitalisation, and death [1]. While vaccine supply, distribution, and access were important to enhance community protection, vaccine hesitancy, and confidence were key determinants of vaccine uptake [2]. Vaccine hesitancy is defined as a ‘motivational state of being conflicted about, or opposed to, getting vaccinated; [and] includes intentions and willingness’ [3].

Globally, First Nations peoples face significant social, political, and economic disparities which can increase COVID-19-related mortality and hospitalisation. For example, in Australia, Aboriginal, and Torres Strait Islander peoples in remote communities are at high risk of death and hospitalisation from COVID-19 due to a high burden of chronic disease, high mobility between communities, inadequate housing conditions, and lack of access to healthcare [4–6]. This is similar to the USA, where COVID-19 disproportionately affected racial and ethnic minorities, including First Nations peoples with a higher risk of severe illness, hospitalisation, and death [7–9]. Increasing COVID-19 vaccination uptake, including receipt of booster doses, reduces the risk of severe illness, hospitalisation, and death.

Vaccine hesitancy contributed to lower vaccine uptake within some First Nations peoples [10]. For example, in 2021 as the COVID-19 vaccination programme was being first rolled out, a survey indicated that a lower proportion of Aboriginal and Torres Strait Islander peoples intended to get the vaccine (37.5%) than non-Indigenous peoples (57.1%) [11]. The survey found that a combination of mistrust in government, community fears over vaccine safety, and exposure to misinformation were likely factors contributing to lower vaccination uptake [12–17]. Australian First Nations peoples’ experiences with vaccine hesitancy are echoed in the USA, where surveys have found that American Indian/Alaskan Native peoples reported lower trust in the COVID-19 vaccine compared to the general population [18–20]. Studies suggested that vaccine hesitancy or refusal was influenced by unreliable information, historical mistrust of medical professionals, desire for autonomy, and fear of political or pharmaceutical industry influences [21]. Similarly, in New Zealand, respondents who identified as Māori have reported lower COVID-19 vaccine acceptance rates [22]. In Canada, the Royal Society of Canada Working Group on COVID-19 Vaccine Acceptance also indicated that vaccine confidence amongst Indigenous peoples in Canada is complex, with distrust linked to the violence of colonialism [23].

These factors are rooted in the colonial history of some countries that has shaped the discrimination of First Nations peoples experience today. The relationship between First Nations peoples and colonising governments has been fraught at times because of policies designed to assimilate them into white society, such as the Stolen Generations in Australia (the practices of removing Aboriginal children from their parents and placing them with white families or institutions in an effort to achieve ‘assimilation’) [15–17]. Colonial mistreatment has also profoundly affected health disparities in other countries, where historical injustices, including forced displacement, cultural assimilation policies, and systemic racism, have led to long-lasting socioeconomic and health inequalities [18,19]. These injustices have fostered a pervasive mistrust in healthcare systems and institutions, which have struggled to provide adequate and culturally sensitive care [18,19].

These injustices are the backdrop against which we locate this systematic review that aimed to examine strategies to address COVID-19 vaccine hesitancy among First Nations peoples. It identified strategies to address vaccine hesitancy, examined delivery methods and formats and investigated if and why certain strategies were successful. We did not use the term ‘vaccine hesitancy’ as a way to blame individuals for their doubt, but respectfully, acknowledging the complex origins of COVID-19 vaccine hesitancy in First Nations peoples, along with efforts to address it. As First Nations peoples are often disproportionately affected by infectious diseases, such as COVID-19, it is important to understand the strategies to address vaccine hesitancy among First Nations peoples. Lessons learned from the COVID-19 pandemic can, and should inform future pandemic planning efforts.

## Methods

An initial rapid review of strategies to address COVID-19 vaccine hesitancy among First Nations peoples was conducted in December 2021-February 2022, with findings reported in February 2022 [20]. This subsequent systematic review was undertaken to synthesize all relevant studies between 2020 and 2023. During the pandemic, a review team was assembled containing members with a variety of methodological and content expertise. A key review member (KC), who has extensive knowledge of administering, managing, and strategically planning culturally safe, strength-based immunisation programmes for Aboriginal and Torres Strait Islander peoples was involved to ensure that First Nations views were represented throughout the review. The protocol for this review was prospectively registered in PROSPERO [CRD42022350739].

### Information sources

The following key bibliographic databases were searched by an information specialist (CK) to locate items for this review: OVID MEDLINE (including Epub Ahead of Print, In-Process & Other Non-Indexed Citations) (1946–23 August 2023), OVID EMBASE (1974–28 August 2023), OVID Global Health (1910-Week 34 2023), OVID PsycINFO (1806-August Week 3 2023), CINAHL via EBSCO (1982–1 September 2023) and the Informit Health Collection September 2023 (including A+Education, AGIS Plus Text, Asia Collection, Australian Public Affairs (APAFT), Business Collection, EduTV, Engineering Collection, Families & Society Collection, Health Collection, Humanities & Social Sciences Collection, Indigenous Collection, Literature & Culture Collection, New Zealand Collection and TVNews).

As per suggested practice [24], a series of initial scoping searches were undertaken and the retrieval from these was assessed to locate additional searching terms. This process identified the specific First Nations peoples terms used in the review, which were used in combination with broader terms representative of all First Nations peoples to ensure comprehensive retrieval. Search terms included both controlled vocabulary terms (where available) and textwords representing the following key conceptual domains: COVID-19/SARS-COV 2, Immunization, Vaccines, Patient Acceptance of Health Care, Health Knowledge, Attitudes and Practice, Vaccination Refusal, Health Promotion, Health Education, Patient Education, Continuing Education, Communication, Leadership, Peer Influence, Indigenous Health Services and Government and Healthcare Financing. For this study, First Nations peoples refer to Indigenous communities that predate colonial influences on their territories globally. These communities are characterised by unique cultural traditions, languages, and historical continuity with their ancestral lands. A broad approach to searching was used to account for over 5000 distinct Indigenous groups across the world. The full search strategy including all variant terms used is available in Appendix A. No language limits were applied. The searches were date limited from 1 January 2020.

The reference lists of included articles were also searched to identify any additional items for inclusion. Grey literature reports and key websites were identified through Google Scholar and in consultation with content experts. The final search was conducted on 1 September 2023.

### Study selection (inclusion/exclusion)

Before starting the article selection process, duplicate citations obtained from different databases were removed. Items were included in this review if they included primary data that discussed COVID-19 vaccine hesitancy in First Nations peoples combined with strategies used to tackle COVID-19 vaccine hesitancy within the defined populations. All study types were included. No language limits were applied, and any non-English items were translated using Google Translate. No country limits were applied.

Studies were excluded if they did not discuss COVID-19 vaccine hesitancy, First Nations peoples or strategies used to address vaccine hesitancy. Descriptive studies that looked at risk factors for hesitancy and provided recommendations about possible strategies but did not implement strategies were excluded. Commentaries, letters, and news items were excluded. Studies were only included if they contained sufficient data for extraction.

### Data collection process

Two independent screeners (AT and MR) screened titles and abstracts against the study selection criteria. Full-text papers of citations of interest were then read to select papers suitable for inclusion. Two independent reviewers (AT and MR) screened the items and independently recorded reasons for inclusion and exclusion. Individual judgements and disagreements were discussed and resolved in regular meetings between AT, MR, CK, and KC.

### Data extraction

Two researchers (AT and MR) independently extracted the following data from the studies: First author, year of publication, publication date, the study aims, study design, study setting, study population, the date range for study data collection, strategies, the author identified limitations, and funding source. Snowballing to identify further studies for inclusion was conducted. Extracted data were tabulated, and results were presented as reported in the studies.

### Quality assessment

The quality of all included studies was initially assessed by one reviewer (AT) using the Joanna Briggs Institute Critical Appraisal tools appropriate to the methodologies used in the included studies [25]; the checklist for qualitative research, quasi-experimental studies, and textual evidence: narrative [21]. This was further reviewed by two other reviewers (MR and CK). Data were not excluded based on study quality.

### Data analysis

All data were synthesised narratively to identify strategies for addressing COVID-19 vaccine hesitancy, specifically within First Nations populations. One reviewer (AT) conducted data analysis in consultation with two reviewers (MR and CK). JL assisted with initial review of themes. KC and JL provided further feedback, oversight, and inputs into the analysis.

## Results

### Identification and selection of studies

**Figure 1.**
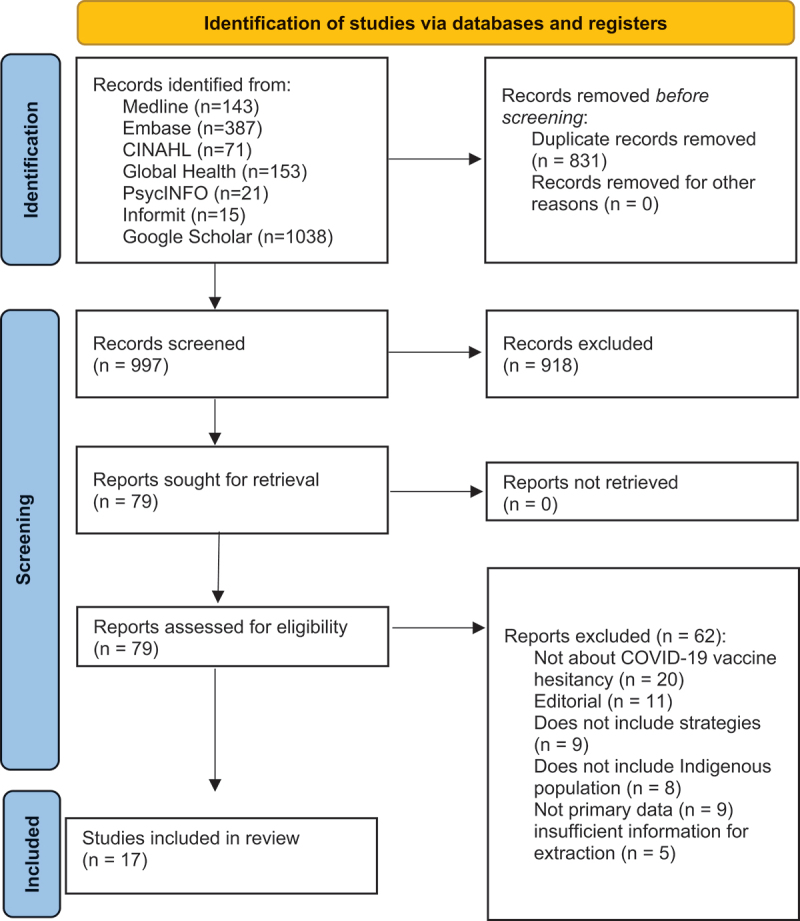
Flow diagram of the selection process.

The search yielded 1,828 records; of these 831 were duplicates and subsequently removed. The remaining 997 records were screened, and 918 records were excluded. We assessed the full text of 79 articles, with 62 excluded after applying inclusion and exclusion criteria. There were 17 included studies. As per PRISMA guidelines, the flow diagram is shown in Figure 1.

### Characteristics of the studies

Of the seventeen included studies, nine were from the USA, three were from Australia, three were from Canada, and two were from Guatemala. Four were mixed-methods studies, three were observational studies, seven were qualitative studies, two were pre-post studies, and one was a case study. These are summarised in Table 1.

**Table 1.** Summary of studies.

Authors	Year	Country	Aims	Study design	Study population	Study settings	Data collection	Strategies
AuYoung et al.	2022	USA	To demonstrate successful approaches in communicating with diverse communities and address challenges for COVID-19 vaccination uptake.	Mixed-methods	Latino/x, Black/African Americans, Native Hawaiian and Pacific Islanders, Indigenous populations, Asian Americans and people in high-risk occupations.	Urban and rural	September 2020	Information gathering with bidirectional communication methods. Outreach in-person and via telephone using existing networks. Communication using trusted messengers in partnership with communities.
Aylsworth et al.	2022	Canada	To investigate challenges and solutions for obtaining COVID-19 vaccines among ethnic minorities in Canada.	Qualitative study	Ethnic minorities groups groups (Arab, Black, East Asian, Latin American, South Asian, and West Asian) or Indigenous identities (First Nations, Métis, and Inuit Peoples within Canada).	Unspecified	Spring 2021 (Canada)	Enhancing accessibility of information by offering translations of important information to Indigenous languages.
Blaxland et al.	2021	Australia	To offer evidence that backs community and other approaches using strength-based approaches among First Nations peoples in Australia.	Qualitative method	People who identify as Aboriginal or Torres Strait Islander in the urban study area.	Urban	February 2021	Vaccine messages specific to Aboriginal audiences that focused on: - Building confidence in the scientific technology of the vaccine - Clarifying the viral genetic material used in the vaccine - Providing accurate information about the risk of vaccine side effects.
Chan et al.	2022	USA	To assess the feasibility of using various community interventions to increase COVID-19 vaccine uptake.	Observational study	Unvaccinated/partially vaccinated Indigenous employees in the state’s health system.	Unclear	August 2021-October 2021	Vaccine education and outreach team with similar cultural backgrounds. Virtual town halls to provide credible information and allow for questions to be answered. Staff huddles to educate employees, with departments with low vaccination rates identified and targeted. Written educational material to address concerns raised in the town hall. Vaccination stations with walk-up options.
Epperson et al.	2022	USA	To investigate COVID-19 vaccine decision-making with First Nations peoples in Los Angeles to gain deeper insights into vaccine perceptions and formulate effective strategies.	Qualitative study	Native American residents in Los Angeles County. Prioritised outreach to and recruitment of individuals from groups with high risk of COVID-19 morbidity and mortality.	Urban	3 December 2020–21 January 2021	Tailoring information and outreach for urban Native American communities.
Fredericks et al.	2022	Australia	To explore the influence of conspiracies on vaccination rates among some First Nations peoples in Australia and find effective ways to stop misinformation.	Mixed-methods	Indigenous and non-Indigenous stakeholders from the Indigenous health sector and Aboriginal Controlled Community Health Organisations.	Urban	2021	Collaborating with trusted community sources to disseminate credible information. Supporting Indigenous workforce with greater resourcing. Strengthening interagency collaboration. Addressing systemic, underlying social and health gaps. Ensuring Indigenous representation across all levels overseeing the implementation of strategies.
Ignacio et al.	2022	USA	To describe the outcome of community engagement for COVID-19 vaccination using focus groups to understand vaccine hesitancy and confidence.	Mixed-methods	Adults aged 18 years or older who identify as a member of the African-American/Black American, Indian/Alaska Native, and Hispanic/Latinx communities residing in Arizona.	Urban and rural	February 2021- August 2021	Using community-based testimonials from community and religious leaders who have received the COVID-19 vaccine. Tailoring messaging for each community. Grounding vaccine messages in the acknowledgement of harmful legacies from the past and current medical and research-related abuses.
Kerrigan et al.	2023	Australia	To investigate the methods used to produce COVID-19 videos for First Nations peoples and provide an assessment of their effectiveness.	Qualitative study	First Nations leaders and Elders in Northern territory. Clinical professionals, health promotion workers, interpreters, artists, language centre workers, members of an Infectious Diseases Indigenous Reference Group, and First Nations trainees aged between 19 and 26 years old.	Urban and rural	3 March 2021–28 October 2021	Messages developed were tailored to address First Nations perspectives. Ensuring information provided was consistent across diverse communities. Tailored briefing documents in plain English. Providing locally relevant vaccine resources, in eight languages, with speakers who were recognisable to the community. Co-design process with Elders in the development and review of materials. Materials distributed through community partnership.
King et al.	2022	Canada	To explain the strategy employed in a collaboration between a provincial healthcare provider and an immunisation clinic led by Canada’s First Nations peoples.	Observational study	First Nations communities and Métis settlements.	Urban	March 2021	Virtual community forum for information and question-answer session with Indigenous health professionals. Local Indigenous-led COVID-19 vaccine clinic with low-barrier and locally controlled.
Le-Morawa et al.	2022	USA	To describe the COVID-19 epidemic and vaccine rollout of the San Carlos Apache Tribe.	Observational study	Sam Carlos Apache Tribe.	Rural	May 2020 - February 2021	Preparing in advance for vaccination roll-out prior to vaccine approval. Featuring Tribal leaders who have been vaccinated on social media. Delivering educational materials in various languages. Enhancing vaccine accessibility through offering different locations.
Miguel et al.	2022	Guatemala	To assess the effect of a social media initiative on vaccination acceptance in First Nations peoples in rural areas of the Guatemalan Central Highlands.	Pre-post intervention design	Guatemalan Indigenous populations.	Urban and rural	March-April 2022	Using qualitative human-centred design to better understand COVID-19 vaccination barriers. Findings from qualitative research informed development of social media campaign. Narration of videos provided by local voice talent. Community engagement through partnership with Wuqu’ Kawoq communities.
Sears et al.	2022	USA	To assess First Nations peoples’ understanding, familiarity, concerns, and perspectives on COVID-19 vaccine acceptance in Arizona, develop culturally appropriate strategies, and evaluate its impact on awareness, trust, and willingness to vaccinate.	Pre-post test	Community members from three Arizona Native nations.	Urban and rural	April-November 2021	Employing reliable communicators for COVID-19 health information. Employing consensus panel approaches to customise and refine educational resources.
Skinner et al.	2023	Guatemala	To understand the First Nations Maya population’s perceptions of the COVID-19 vaccine in Guatemala’s Central Highlands and explore appropriate strategies to increase uptake.	Qualitative studies	Indigenous men and women and nurses and physicians.	Urban and rural	November 2021	Story telling by vaccinated community members to enhance vaccine safety. Appropriate religious and cultural messages employed using trusted faith and religious leaders. Linguistically and culturally appropriate messages with tailored images for First Nations communities. Appealing messages designed to resonate with individuals with limited literacy skills. Using trusted sources for health messaging for Indigenous community members.
Sullivan et al.	2023	Canada	To explore vaccine hesitancy among First Nations peoples in Saskatchewan, strategies to address it, and the impact of historical colonialism and contemporary societal influences.	Mixed-methods	First Nations community in Saskatchewan.	Unclear	January and February 2022	Engaging community Elders to participate in ceremonial blessings for the vaccine. Partnering with communities to advocate for vaccination and co-create messaging strategies. Supporting Indigenous communities in developing their own solutions. Creating messaging that is not polarising vaccine confident and vaccine hesitant people.
Tutt et al.	2022	USA	To develop intervention that was designed to utilise trusted health messengers in Navajo Nation as an effective means to address COVID-19 vaccine concerns and hesitancy.	Qualitative study	Native American residents in Los Angeles County. Prioritised outreach to and recruitment of individuals from groups with high risk of COVID-19 morbidity and mortality.	Rural	N/A	Employing a consensus panel comprising of community health representatives and Indigenous students to create and evaluate education materials. Educating Indigenous college students with culturally relevant materials to empower them as health advocates delivering vaccine safety information.
Wong et al.	2023	USA	To describe the partnership between the Mashpee Wampanoag Tribe and CDC I epidemiology, case probing, contact tracing, infection prevention, community prevention tactics, and vaccination efforts.	Case Study	Mashpee Wampanoag Tribe.	Unclear	June 2020-December 2021	Starting preparatory measures to enhance vaccination initiatives and public awareness campaigns. Issuing proactive public announcements ahead of vaccine availability. Collaborating with community to conduct COVID-19 vaccine education sessions.
Zhou et al.	2022	USA	To perform formative research to guide the creation of a public health initiative designed to advocate for preventive actions and enhance COVID-19 vaccinations among ethnic minorities in the USA.	Qualitative studies	Participants between 18–89 years old, self-identified as Latino American, African American or Black, American Indian or Alaska Native that speak either English or Spanish.	Urban	29 October and 24 November 2020	Including different cultural practices in prevention messages. Preferencing positive messaging to give hope that pandemic would end eventually. Translating messages into tribal language to make messages more relevant to their culture.

### Principal findings

Five key strategies were identified to address hesitancy and attempt to address COVID-19 vaccination hesitancy and increase vaccine uptake among First Nations communities.

#### Understanding communities’ needs

Implementors gathered information to understand the concerns of communities to tailor strategies. Methods included virtual town halls or community forums that ensured bidirectional communications where attendees could pose questions [22,23,25]; in-depth interviews, or focus group discussions with communities and key groups such as youths and parents [22,26]; surveys of trusted members of the communities; and community meetings or consensus panels [22,27]. For example, in California, a town hall session featured an Indigenous and Native American physician and physician-in-training to answer questions about the COVID-19 vaccines and SARS-CoV-2 variants. Bilingual and bicultural presenters received positive feedback from the community members, who felt comfortable and safe to seek further information because they were communicating in their native language [22]. Similarly in Guatemala, researchers used a qualitative human-centred design approach via key informant interviews and focus groups to better understand COVID-19 vaccination barriers among Guatemalan Indigenous populations. Subsequently, the findings from the research informed the development of a social media campaign [26].

#### Collaborating with communities

The need to collaborate with targeted communities for planning and implementation was evidenced in several studies. Interagency collaboration was noted as a strategy [22,28–31]. Collaborating directly with communities and organisations provided better information access and ensured that strategies could be co-designed appropriately [28]. Community members spearheaded activities to integrate creative, respectful, and appropriate ways of information sharing, which built trust in the vaccine messages [22,32].

For example, in Australia, the National Aboriginal Community Controlled Health Organisation arranged meetings with Aboriginal church leaders to discuss vaccine misinformation and strategies to unite faith-based medical messaging [29]. In California, trusted messengers communicated with the community in person and via phone through existing networks to share resources and information, demonstrate that vaccines are part of the pandemic mitigation plan, and help make vaccine appointments [22]. In Massachusetts, the US Centers for Disease Control and Prevention (USCDC) and community leaders from the Mashpee Wampanoag Tribe worked together in organising and running a COVID-19 vaccine information session to tackle misinformation and misunderstanding [28]. In Arizona, researchers worked together with Indigenous college students to provide training with culturally centred materials to become health messengers providing vaccine safety education [27].

Another strategy was ensuring that vaccination clinics were locally available and controlled by the community to ensure a respectful, and culturally appropriate vaccination experience [25,28]. For example, in Alberta, Canada, COVID-19 vaccine clinics were led by M*é*tis health professionals and were locally controlled by this community. M*é*tis health professionals were available throughout the clinics to answer questions and ensure the vaccination clinics were culturally safe [25]. Communities highlighted that the provision of a locally run COVID-19 vaccine clinic made a positive contribution to vaccine-decision-making [25].

#### Tailored messaging

Tailored messaging within vaccination campaigns was an integral strategy highlighted within the studies. Tailored messaging focused on vaccine acceptability, efficacy, and safety by providing accessible scientific information [32]. Testimonials from trusted community members such as local leaders, elected officials, elders, religious leaders, or other community members who received the COVID-19 vaccine substantiated this messaging [22,23,28,33]. Messaging strategies also addressed any practical barriers to vaccination experienced by the community, including eligibility, access, and cost [32].

Implementors also tailored the content of the messaging according to the needs and preferences of different First Nations peoples. This involved using familiar faces, cultural practices, and cultural references integrated into the messaging [22,34]. This also included dissemination using trusted health messengers or using media platforms that resonated with the community. A common strategy was to create messaging in the language of the First Nations communities [31,34–36]). Different modes of communication were used, with audience segmentation to tailor messaging according to the needs and preferences of different First Nations communities [23]. For example, in Alberta, Canada, question-and-answer sessions were held with M*é*tis health professionals as trusted messengers in the community [25]. Messages from First Nations health professionals which were tailored to specific regions or settlements were an effective means to disseminate credible and appropriate information about the vaccine [25].

#### Acknowledging underlying systemic traumas and social and health gaps

First Nations peoples are disproportionately affected by chronic disease, inadequate housing conditions, and a lack of access to healthcare. Historical and contemporary abuse and trauma experienced by First Nations peoples has contributed to a culture of mistrust and vaccine hesitancy [28,33]. Thus, any strategy should include consideration of the experiences of racism and historical mistreatment of First Nations peoples in the active colonisation process. One strategy highlighted by an included study in Aboriginal communities in New South Wales (NSW) was anchoring vaccine messages in the acknowledgement of the harmful legacies from past and current medical and research mistreatments that communities may have experienced [33]. This was echoed in another study in Western Australia whereby a First Nations community leader talked through community concerns in a manner that acknowledged the impact of trauma from historical policies such as the Stolen Generations and the understandable mistrust that has come from it while reiterating the importance of COVID-19 vaccination [28].

#### Early logistics planning

Along with other interventions, two studies noted effective planning of interventions and logistics as a strategy. They described how careful planning is needed to ensure that strategies with the aim of reducing vaccine hesitancy are developed and ready for when the roll-out begins, and that planners anticipate operational issues that may otherwise hinder or create difficulty in accessing vaccination [29,35]. Wong et al. described how the Mashpee Wampanoag Tribe collaboration with the USCDC involved early planning between tribal, local, and state vaccination activities. They pre-emptively released public statements and began their campaign before the COVID-19 vaccine was available, thereby pre-empting vaccine hesitant messaging [29]. A similar strategy was described in Le-Morawa et al., where there was advance planning for the vaccine roll-out before it was approved. Community education began six weeks before the COVID-19 vaccine introduction in the San Carlos Apache Tribe [35]. Services were made more convenient through the use of on-site, drive-through, pop-up, and door-to-door vaccination [35].

## Discussion

This systematic review synthesised available evidence of strategies to address hesitancy and increase COVID-19 vaccination uptake among First Nations peoples. To our knowledge, this is the only systematic review examining this topic and builds on our initial rapid review. We identified several strategies across Australia, the USA, Canada, and Guatemala: understanding communities’ needs, collaborating with communities, tailored messaging addressing underlying systemic traumas and social health gaps, and early planning and early logistics planning. This review provides valuable insights into strategies policymakers, practitioners, and community leaders can implement to work with First Nations peoples for the long-term management of COVID-19 and other new vaccine programmes in the future. It is particularly relevant to pandemic planning and could be considered as a form of community engagement as per the activities of Joint External Evaluations [37] and Global Preparedness Monitoring Board [38].

Importantly, the findings in this review highlight the need to address the systemic causes of health inequities that resulted in First Nations peoples’ vulnerability to the disease and vaccine hesitancy [39]. Stemming from the longstanding and continuing impacts of colonisation, physical, social, and mental health inequalities are amplified by the exclusion of First Nations peoples from mainstream health services [40]. Even prior to the pandemic, First Nations peoples often reported being unable to access adequate healthcare or have their health needs effectively met [25,41]. Addressing systemic issues and building trust in health services is key to improving health outcomes and ensuring the safety of First Nations peoples in health emergencies. Strategies to address hesitancy, often focusing on good communication, must also go hand-in-hand with those that ensure services are accessible and culturally respectful. Good communication should prioritise transparency, empathy, and respect for patient autonomy. Providing clear, evidence-based information while responsively addressing concerns can enhance trust and acceptance [42,43].

Indigenous-controlled healthcare services support this approach. They have played an important role in the treatment and management of disease, prevention, and health promotion, and addressing the social determinants of health to redress existing health inequities [25,28,39]. This service delivery model is characterised by accessible health services, community involvement, a culturally appropriate and skilled workforce, self-determination, and empowerment, along with multidisciplinary collaboration [41]. This requires the investment of time, attention, and funding to ensure sufficient and sustainable resources to strenghten existing capacity in First Nations peoples [44].

Ensuring that First Nations peoples are empowered to have self-determination in healthcare decisions is key among the strategies identified through the systematic review, as it is a cross-cutting theme across the strategies. Locally led, holistic, comprehensive, tailored, and culturally appropriate strategies have effectively increased vaccination coverage among First Nations peoples [22–25,45,46]. Leadership from First Nations peoples has promoted better outcomes for First Nations peoples [22,23]. Such leaders are best placed to recognise the needs of the communities they serve, and to decide on, and develop culturally appropriate and effective strategies to increase vaccination coverage [45,46]. Further, they are also more likely to be able to mobilise support from their communities [45,46]. It is also important to couple self-determination with appropriate resourcing and equitable government policies [45].

Integral to the abovementioned strategies is the government’s logistics planning. It is important to have early planning of the vaccine roll-out, combined with advanced preparation of messaging and community education. Issues around vaccine access and poorly planned vaccination campaigns invariably impact vaccine acceptance, as they allow for other messages to fill the void [47]. Barriers to vaccine access can be tackled in advance by implementing approaches to ascertain the unique barriers to vaccination experienced by First Nations peoples early in a vaccination campaign [47].

### Recommendations

The COVID-19 pandemic presented considerable risk to First Nation peoples. Continued investment in strategies to address vaccine hesitancy will be beneficial for continued efforts in COVID-19 booster uptake [4,5] and to inform future pandemic planning efforts. Lessons learned from the COVID-19 pandemic will also have important implications for how First Nations peoples are supported in future public health crises. Strengthening First Nations-owned and led strategies to address vaccine hesitancy within their communities will require a paradigm shift to create an enabling environment in policy and practice. We highlight key strategies to support this structural change: active listening, collaboration with community, and tailored interventions, with self-determination as integral throughout.

This study has shown that research and policymaking in relation to First Nations peoples must have an embedded relationship with First Nations peoples and advocates. This can involve a co-designing element implementing two-way communication and engagement with key representatives as partners rather than audiences to persuade or command [48]. Embedding First Nations voices in organisational decision-making is key to ensuring the longer-term goal of First Nations peoples in positions of power in government and health. One recent documented model in Australia involved reconstructing existing governance models to allow for respectful and meaningful space for Aboriginal peoples to co-design and co-share the governance of health service delivery [49]. Governments should also continue to invest in capacity building in First Nations health workforce. This could involve school-based traineeships and providing scholarship opportunities for university degrees. It could further involve supported processes for a more streamlined application.

Lastly, it will be important to ensure that lessons learned from implementing strategies can be documented, evaluated, and disseminated using First Nations-centred approaches [50,51]. Indigenous data sovereignty should also be placed at the heart of dissemination processes to ensure that the needs of First Nations stakeholders and practitioners are appropriately considered [52]. Many strategies to address vaccine hesitancy take place in community-based settings. Health practitioners are often met with challenges in writing for publication which can limit the reach of lessons learned [53–55]. Collaboration with academic institutions may help to build capacity and support dissemination processes, along with having sufficient time to do so.

Central to all strategies is to both acknowledge and address the underlying systemic traumas and social and health gaps in the design of strategies to overcome vaccine hesitancy. Health systems issues must be addressed so that First Nations peoples are not disproportionately affected by COVID-19 or other emerging infectious diseases.

#### Strengths and limitations

A strength of this review is that First Nations perspectives ground the findings and recommendations. At the time of writing this review, KC was the National Indigenous Immunisation Coordinator at the National Centre for Immunisation Research and Surveillance (NCIRS), Australia. KC has extensive knowledge of administering, managing, and strategically planning immunisation programmes for Aboriginal and Torres Strait Islander peoples in Australia. KC has expertise in programmes that benefit Aboriginal and Torres Strait Islander peoples using community methodologies and qualitative research methodologies.

A limitation of this review is that our inclusion and exclusion criteria limit the findings to peer-reviewed and grey literature. The emergency nature of the COVID-19 vaccination roll-out may have affected the time and ability of researchers, policymakers, and implementers to write publications that fit within the systematic review methodology [53]. Strategies not written into a peer-reviewed publication or grey literature reports may have been developed and employed. Thus, whilst we employed a systematic approach to best find relevant studies, there may be other undocumented strategies to reduce vaccine hesitancy in First Nations peoples. Further, despite a robust search approach including the use of variant search terms, specific terms for every one of the over 5,000 First Nations peoples globally were not included, thus the study may possibly may have missed articles that did not define the study population broadly within the search terms set out in Appendix A. It is also possible that First Nations peoples are not a policy priority in certain countries, and therefore studies are not conducted or published.

The review found mostly observational and qualitative studies. These provided valuable insights, often rich in detail and context, but were less able to quantitatively evaluate the effectiveness of interventions. The heterogeneous nature and methodological limitations of these studies may restrict the extent to which conclusions can be generalised. The heterogeneity of First Nations peoples limits generalisation because, despite shared experiences of colonisation, each community maintains its distinct identity, stories, strengths, and challenges. Recognising this diversity is crucial for understanding the unique cultural, social, and historical contexts of First Nations peoples globally. Nevertheless, the findings tie in with broader evidence on strategies to address hesitancy. It finds that, while direct communication or informational interventions alone have limited effects on uptake, community engagement is effective. Moreover, such an approach is imbued with justice, respect, and self-determination, which are key elements of justice for First Nations peoples.

## Conclusion

The inclusion of First Nations-centred strategies to reduce COVID-19 vaccine hesitancy is essential to ensuring an equitable pandemic response. This systematic review found a common set of strategies around understanding communities’ needs, collaboration with communities, tailored messaging, logistics planning, and addressing the underlying systemic traumas experienced by First Nations peoples when accessing healthcare. Implementing these strategies in the continued effort to vaccinate against COVID-19 and potentially for other outbreaks is integral to ensuring that First Nations peoples are not disproportionately affected by infectious diseases. These strategies should be considered in future pandemic planning efforts.

## Appendix A: Ovid MEDLINE search strategy

Database: MEDLINE(R) All including Epub Ahead of Print, In-Process & Other Non-Indexed Citations, Daily and Versions(R) <1946-current>

Search Strategy:

——————————————————————————–

1 exp COVID-19/
2 exp SARS-CoV-2/
3 (‘2019 nCoV’2019 nCoV$’#8217; or 2019-nCoV$ or 2019nCoV$ or ‘n CoV‘n CoV$’#8217; or n-CoV$ or nCoV$).tw.
4 (‘covid 19’ or COVID-19 or covid19).tw.
5 (“Severe Acute Respiratory Syndrome Coronavirus 2” or “Severe Acute Respiratory Syndrome Coronavirus-2” or “SARS coronavirus 2” or “SARS coronavirus-2”).tw.
6 (‘SARS CoV2’ or SARS-CoV2 or SARSCoV2 or SARS-CoV-2).tw.
7 1 or 2 or 3 or 4 or 5 or 6
8 exp Immunization/
9 exp Immunization Programs/
10 exp Vaccines/
11 (immunis$ or immuniz$ or vaccin$).tw.
12 8 or 9 or 10 or 11
13 7 and 12
14 exp COVID-19 Vaccines/
15 13 or 14
16 exp ‘Patient Acceptance of Health Care’/
17 exp Health Knowledge, Attitudes, Practice/
18 (attitude$ or knowledge$ or belie$ or view$ or opinion$ or thought$ or think$ or perceive$ or percepti$ or perspective$ or understand$ or prefer$ or practice$ or behav$ or trust$).tw.
19 exp Vaccination Refusal/
20 (hesitan$ or confiden$ or accept$).tw.
21 (strateg$ or plan$ or initiative$ or interven$ or effort$ or approach$ or polic$ or program$ or project$ or proposal$ or system$ or method$ or scheme$ or arrang$ or incentiv$ or implement$).tw.
22 exp Health Promotion/
23 exp Health Education/
24 exp Patient Education as Topic/
25 exp Education, Medical, Continuing/
26 (cme$ or cpd$).tw.
27 (promot$ or educat$ or inform$ or teach$ or taught).tw.
28 exp Communication/
29 communicat$.tw.
30 (messag$ or frame$ or framing or dialogue$).tw.
31 exp communications media/
32 exp video-audio media/
33 exp Webcasts as Topic/
34 (written or write or writing or text$ or audio$ or video$ or image or images or visual or visuals or visually or animat$ or cartoon$ or graphic$ or tweet$ or post or posts or posting).tw.
35 (radio or radios or televis$ or tv or media or webpage$ or print$ or email$).tw.
36 (text adj1 messag$).tw.
37 (social adj1 media$).tw.
38 (podcast$ or webcast$ or webinar$ or broadcast$ or advertis$ or pamphlet$ or brochure$ or news or article$).tw.
39 inform$.tw.
40 yarn$.tw.
41 setting$.tw.
42 (safe$ adj4 place$).tw.
43 (clinic or clinics).tw.
44 exp Leadership/
45 (leader$ or champion$ or advocate$).tw.
46 exp Peer Influence/
47 peer$.tw.
48 exp Health Services, Indigenous/
49 exp Financing, Government/
50 exp Healthcare Financing/
51 (financial$ or fund$ or pay$ or paid).tw.
52 cash$.tw.
53 free$.tw.
54 reimburse$.tw.
55 allowance$.tw.
56 bonus$.tw.
57 16 or 17 or 18 or 19 or 20 or 21 or 22 or 23 or 24 or 25 or 26 or 27 or 28 or 29 or 30 or 31 or 32 or 33 or 34 or 35 or 36 or 37 or 38 or 39 or 40 or 41 or 42 or 43 or 44 or 45 or 46 or 47 or 48 or 49 or 50 or 51 or 52 or 53 or 54 or 55 or 56
58 15 and 57
59 exp Indigenous Peoples/
60 indigenous.tw.
61 (aborigin$ or torres strait or koori$).tw.
62 maori$.tw.
63 exp ‘Native Hawaiian or Other Pacific Islander’/
64 exp Indians, North American/
65 exp Indigenous Canadians/
66 (eskimo$ or inuit$ or metis or aleut$ or american indian$ or native american$ or tribal$).tw.
67 (first adj1 (nation or nations)).tw.
68 (first adj1 people$).tw.
69 59 or 60 or 61 or 62 or 63 or 64 or 65 or 66 or 67 or 68
70 58 and 69
71 limit 70 to yr=‘2020 -Current’
